# Investigation of novel alkyl/benzyl (4-sulphamoylphenyl)carbamimidothioates as carbonic anhydrase inhibitors

**DOI:** 10.1080/14756366.2022.2152811

**Published:** 2023-01-11

**Authors:** Morteza Abdoli, Alessandro Bonardi, Claudiu T. Supuran, Raivis Žalubovskis

**Affiliations:** a Institute of Technology of Organic Chemistry, Faculty of Materials Science and Applied Chemistry, Riga Technical University, Riga, Latvia; b Neurofarba Department, Universita Degli Studi di Firenze, Florence, Italy; c Latvian Institute of Organic Synthesis, Riga, Latvia

**Keywords:** Carbonic anhydrase, inhibitors, sulphonamides, carbamimidothioates, bacterial enzymes

## Abstract

A library of novel alkyl/benzyl (4-sulphamoylphenyl)carbamimidothioates was synthesised by selective *S*-alkylation of the easily accessible 4-thioureidobenzenesulphonamide. The compounds were assayed as inhibitors of four human (h) carbonic anhydrase isoforms hCA I, II, VII, and XIII, as well as three bacterial enzymes belonging to the β-CA class, MscCA from *Mammaliicoccus* (*Staphylococcus*) *sciuri* and StCA1 and StCA2, from *Salmonella enterica* (serovar *Typhimurium*). Most compounds investigated here exhibited moderate to low nanomolar inhibition constants against hCA I, II, and VII. The cytosolic hCA XIII was also inhibited by these compounds, but not as effective as hCA I, II, and VII. Several compounds were very effective against MscCA and StCA1. StCA2 was less inhibited compared to MscCA and StCA1. Some compounds showed considerable selectivity for inhibiting some CA isoforms. They may thus be considered as interesting starting points for the discovery and development of novel therapeutic agents belonging to this class of enzyme inhibitors.

## Introduction

Enzymes are drug targets for at least 50% of the clinically used drugs ^1^. At leasta third of all known enzymes contain one or more metallic ions (such as Ca, K, Mg, Mn, Fe, Co, Ni, Cu, Zn, and Mo), that are essential to their biological functions^2^^,^^3^. Among the zinc-containing metalloenzymes, the carbonic anhydrases (or carbonate dehydratases) (CA, EC 4.2.1.1) are abundantly present in most life forms, e.g. mammalian tissues, plants, algae as well as all types of microorganisms, such as bacteria, protozoans, diatoms, etc^4^. Human CAs occur in at least 15 isoforms (CA I, II, III, IV, VA, VB, VI, VII, VIII, IX, X, XI, XII, XIII, and XIV) depending upon the tissue, organ, or cell-type they are distributed in^5^. This enzyme assists the chemical interconversion of carbon dioxide and water to bicarbonate and H^+^ ions, which are thereafter involved in mammals in both physiological and pathological processes, including respiration, pH homeostasis, signal transduction, electrolyte secretion, bone resorption, lipogenesis, calcification, etc^6–8^. Due to broad roles of carbonic anhydrases in metabolism, selective inhibition of their activity is an excellent therapeutic target for several diseases^5^, including glaucoma (CA II, IV), edoema (CA II), obesity (CA VA), cancer (CA IX, XII), sterility (CA XIII), haemolytic anaemia (CA I), altitude sickness (CA II), and more recently in the management of neuropathic pain (CA VII) or as antiinfectives (when pathogenic organisms CAs are being targeted)^9^.

**Scheme 1.**
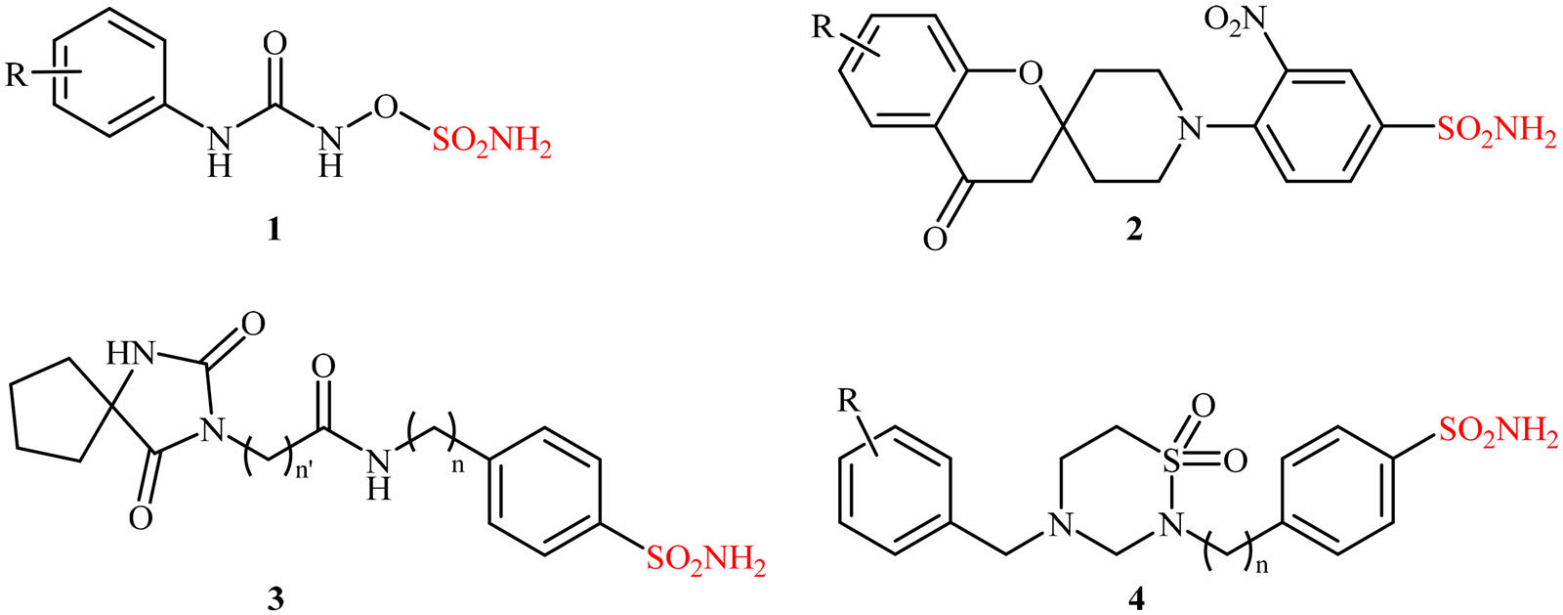
Chemical structures of some selected examples of effective CA inhibitors **1–4**.

Sulphonamide-containing compounds are the main class of CA inhibitors^5^. More than 70 currently marketed drugs contain this privileged moiety^10^, and many researchers have been working to explore it, due to the potential of sulphonamides against diverse diseases. In Scheme 1, some of the effective CA inhibitors of the sulphonamide/sulphamate types (compounds **1–4**) are shown.

In continuation of our work on the developments of carbonic anhydrase inhibitors^11^, in this study we report the synthesis of a panel of 15 novel alkyl/benzyl (4-sulphamoylphenyl)carbamimidothioates, for which we explored their inhibitory effects against a panel of human and bacterial CA isoforms involved in various pathologies.

## Results and discussion

### Chemistry

**Scheme 2.**
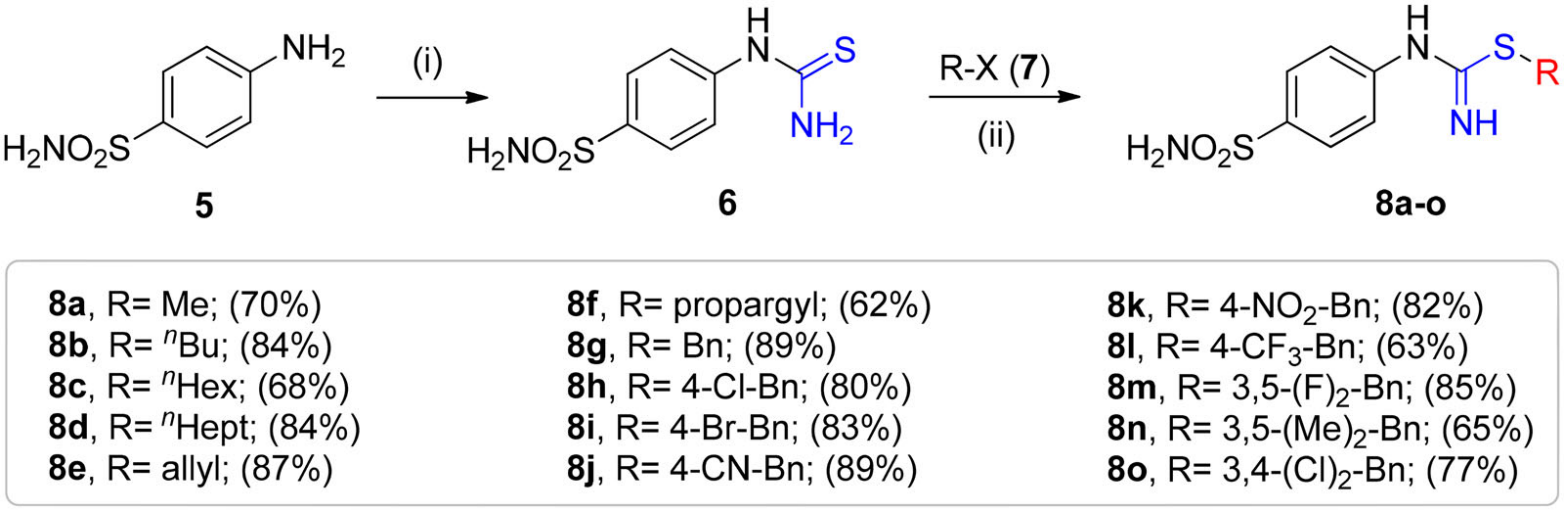
Reagents and conditions: (i) KSCN, aq. 3.5 M HCl, reflux, 3 h, 31%; (ii) DMF 20–100 °C, 2.5–24 h.

The synthetic route to obtain the target compounds reported here is outlined in Scheme 2. The 4-thioureidobenzenesulphonamide intermediate **6**, prepared according to the literature procedure^12^^,^^13^ was upon treatment of sulphanilamide **5** with KSCN in a refluxing aqueous solution of HCl. The target alkyl/benzyl (4-sulphamoylphenyl)carbamimidothioates **8a–o** were selectively synthesised by reaction of 4-thioureidobenzenesulphonamide **6** with the corresponding alkyl/benzyl halides **7** at elevated temperatures. Except for a few compounds which were purified by column chromatography, most of them were purified by simple extraction using ethyl acetate, which afforded the desired products with high purity and yields ranging from 62% to 89%. The analytical and spectroscopic data (^1^H and ^13^C NMR chemical shifts and mass spectra) of the purified compounds are in agreement with the purposed structures (see Experimental section for details).

### Carbonic anhydrase inhibition

Sulphonamides **8a–o** incorporating carbamimidothioate moieties have been tested as inhibitors of four human (h) CA isoforms, the cytosolic hCA I, II, VII and XIII, as well as the bacterial β-CAs from *Mammaliicoccus* (*Staphylococcus*) *sciuri*^14^ and *Salmonella enterica* (serovar *Typhimurium*), StCA1 and StCA2^15^. It should be mentioned that the first bacterial enzyme, MscCA, was originally reported by us as *Staphylococcus aureus* β-CA, SauCA based on a genomic sequence annotated in the data bases in 2017^14^. A recent reanalysis of that sequence revealed that the original annotation was erroneous, and that the sequence encodes a β-class CA from another *Staphylococcaceae* family member, i.e. *Staphylococcus sciuri*, which is a Gram-positive, oxidase-positive, coagulase-negative member of these infectious bacteria known to provoke disease in humans and animals (it was originally isolated from the squirrel)^16^. In 2020, the species was renamed as belonging to a new genus, as *Mammaliicoccus sciuri*^16^. The hCAs included in the study were the ubiquitous hCA I and II, as well as hCA VII and XIII which are found in fewer tissues compared to hCA I and II, and are involved in several pathologies in the CNS (hCA VII) or reproductive tract (hCA XIII)^17^^,^^18^.

The following structure-activity relationship (SAR) can be evidenced from the data of Table 1.

**Table 1.** Inhibition data of human CA isoforms CA I, II, VII, and XIII and bacterial β-CA isoforms MscCA, from *Mammaliicoccus* (*Staphylococcus) sciuri*, and StCA1 and StCA2, from *Salmonella enterica* (serovar *Typhimurium*), with compounds **8a–o** using **AAZ** as standard drug. 
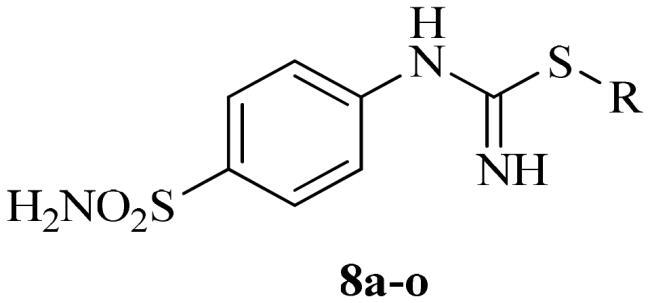

Cmpd	R	K_I_ (nM)^a^						
		hCA I	hCA II	hCA VII	hCA XIII	MscCA	StCA1	StCA2
**8a**	–CH_3_	90.4	85.8	18.9	97.5	759.9	87.4	270.2
**8b**	–(CH_2_)_3_CH_3_	76.4	73.6	13.8	341.6	889.8	91.7	347.8
**8c**	–(CH_2_)_5_CH_3_	78.8	2.5	2.3	92.2	946.7	82.5	897.8
**8d**	–(CH_2_)_6_CH_3_	69.6	9.3	2.7	925.9	3703	71.3	955.3
**8e**	–CH_2_CH = CH_2_	38.5	8.2	1.8	814.4	879.2	313.1	4987
**8f**	–CH_2_C≡CH	92.5	6.9	5.5	351.6	2479	79.8	7121
**8g**	–CH_2_C_6_H_5_	68.1	53.3	2.4	490.7	360.8	52.2	901.7
**8h**	CH_2_(4-Cl–C_6_H_4_)	73.2	34.4	2.0	892.0	618.9	89.7	6680
**8i**	–CH_2_(4-Br–C_6_H_4_)	60.0	29.9	2.1	907.3	8761	750.9	9697
**8j**	–CH_2_(4-CN–C_6_H_4_)	82.8	21.5	1.5	301.6	925.4	55.7	5194
**8k**	–CH_2_(4-NO_2_–C_6_H_4_)	77.2	9.0	2.6	855.7	934.3	86.2	6853
**8l**	–CH_2_(4-CF_3_–C_6_H_4_)	73.5	8.9	2.3	80.5	658.8	73.4	5894
**8m**	–CH_2_(3,5-diF–C_6_H_3_)	82.2	1.7	1.2	75.6	742.3	91.0	6306
**8n**	–CH_2_(3,4-diCl–C_6_H_3_)	40.5	9.4	7.7	69.3	7816	301.2	8705
**8o**	–CH_2_(3,5-diCH_3_–C_6_H_3_)	83.9	23.3	25.6	750.4	8455	281.8	7884
**AAZ**	–	250	12.5	2.5	16.0	625	59	84

^a^Mean from three different assays, by a stopped-flow technique (errors were in the range of ± 5–10% of the reported values).

(i) The slow cytosolic human isoform hCA I was strongly inhibited by all the synthesised carbamimidothioate derivatives with K_I_ ranging between 38.5 and 92.5 nM. These results indicated that all compounds reported here are more potent than the standard drug acetazolamide (AAZ) on this isoform. Overall the relative inhibitory rates of reported carbamimidothioates followed the order: allyl carbamimidothioate ≥ benzyl carbamimidothioates ≈alkyl carbamimidothioates ≥ propargyl carbamimidothioates. Although the SAR for the benzylic derivatives was not flat, the aliphatic derivatives showed a very flat SAR where the inhibition increased as the chains got longer.

(ii) The ubiquitous cytosolic human isoform hCA II was also effectively inhibited by all compounds in low to moderate nanomolar range with K_I_ values ranging between 1.7 and 85.8 nM. Compound **8m** having a 3,5-difluorobenzyl substitution was found to be the most effective amongst all reported derivatives, whereas the least effective inhibitor was the methyl-substituted compound **8a**. The results showed that the presence of electron-withdrawing functionalities on the phenyl ring periphery of benzylic carbamimidothioates increased their inhibitory activities, whereas electron-donating groups reduced their potency. In the cases of aliphatic carbamimidothioates, it seems that the inhibitory activities increased with the increasing length of the carbon chain. For instance, the heptyl-substituted carbamimidothioate **8c** was 34 times more potent as a CA II inhibitor compared to the methyl-substituted analogue **8a**.

(iii) The brain-associated cytosolic isoform hCA VII showed a rather similar inhibition profile with hCA II, with most compounds investigated here. Thus, the 3,5-difluorobenzyl-substituted compound **8m** was the strongest inhibitor with a K_I_ of 1.2 nM whereas 3,5-dimethylbenzyl-, methyl-, and butyl-substituted carbamimidothioates **8o**, **8a**, and **8b**, respectively, showed the weakest inhibitory capacities, with inhibition constants in the range of 13.8–25.6 nM (but they are still highly effective inhibitors). It is worth mentioning that more than half of the compounds investigated here (**8c**, **8e**, **8g**–**8j**, **8l**, **8m**), showed better inhibitory activities against hCA VII in comparison with the standard drug AAZ. Among them, half (**8g**–**8j**) showed more than 10 times a better selectivity against hCA VII vs hCA II.

(iv) The other cytosolic isoform hCA XIII was weakly inhibited by all investigated compounds (K_I_ in the range of 69.3–925.9 nM) compared to AZA, which showed a K_I_ of 16.0 nM. The best inhibitors were the 3,4-dichlorobenzyl- and 3,5-difluorobenzyl-substituted carbamimidothioates, **8n**, **8m**, which were however almost four times less potent inhibitor than acetazolamide.

(v) Against the bacterial β-CA isoform MscCA, except the benzyl- and 4-chlorobenzyl-substituted carbamimidothioates **8g**, **8h**, with K_I_ values of 360.8 and 618.9 nM, respectively, the other compounds acted as weak inhibitors (K_I_ in the range of 658.8–8761 nM) compared to the clinically used sulphonamide acetazolamide. It is noteworthy that while the allylic carbamimidothioate **8e** showed the best result against hCA I, the worst result against MscCA were observed for this derivative. These results clearly indicated that the interaction of inhibitors with α- and β-CAs is rather different.

(vi) The bacterial β-CA isoform StCA1 was also inhibited by the synthesised compounds **8a–8n** in a moderate nanomolar range with K_I_ values of 52.2 to 750.9 nM. Similar to MscCA, the most potent inhibitor against this isoform was compound **8g** with a potency similar to that of AAZ. Interestingly, the less effective inhibitor was again the benzyl-substituted derivative **8i** with 4-bromo substitution. These results revealed that the inhibitory activity of benzyl-substituted carbamimidothioates **8g–8o** against StCA1 significantly depended on the electronic effects of substituent on the phenyl ring. Generally, unsubstituted and strongly electron-withdrawing groups (e.g. CN, NO_2_) at the benzylcarbamimidothioate moiety induced higher inhibitory activities compared to electron-donating (e.g. Me) or less electron-withdrawing groups (e.g. Cl, Br).

(vii) StCA2 was the least inhibited bacterial CA among the three such enzymes investigated in this study. Indeed, the newly prepared compounds showed K_I_ ranging between 270.2 and 9697 nM. Therefore, all of them were generally poor StCA2 inhibitors compared to AAZ. Interestingly, unlike StCA1, for which increasing the carbon chain-length of aliphatic carbamimidothioates led to an increased inhibition, for StCA2, the inhibition decreased by increasing the carbon chain-length in the synthesised derivatives.

## Experimental protocols

### Chemistry

Reagents, starting materials and solvents were obtained from commercial sources and used as received. Thin-layer chromatography was performed on silica gel, spots were visualised with UV light (254 and 365 nm). NMR spectra were recorded on Bruker 300 spectrometer with chemical shifts values (δ) in ppm relative to TMS using the residual DMSO-d_6_ signal (^1^H 2.50; ^13^C 39.52). High-resolution mass spectra (HRMS) were recorded on a mass spectrometer with a Q-TOF micro mass analyser using the ESI technique.

### Synthesis

#### 4-Thioureidobenzenesulphonamide (6)


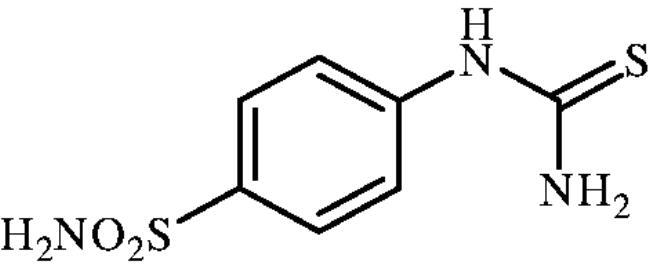


4-Aminobenzensulphonamide (**5**) (30 g, 174.3 mmol) was dissolved in 180 ml of 3.5 M HCl at 70 °C. After cooling to room temperature, KSCN (16.94 g, 174.3 mmol) was added and the mixture was refluxed for 3 h. After cooling to room temperature, the reaction mixture was diluted with ice-cooled water, filtered, washed with water. Solids formed were collected by filtration, washed with water, and air dried to afford **5** (12.1 g, 31%) as white powder.

^1^H NMR (300 MHz, DMSO-d_6_) *δ* = 7.32 (s, 2H), 7.69 (d, 2H, *J =* 8.6 Hz), 7.77 (d, 2H, *J =* 8.6 Hz), 10.02 (s, 1H) ppm ^13^C NMR (75 MHz, DMSO-d_6_) *δ* = 122.8, 127.3, 139.8, 143.9, 182.8 ppm MS (ESI) [M + H]^+^: m/z 232.0.

#### Methyl (4-sulphamoylphenyl)carbamimidothioate (8a)


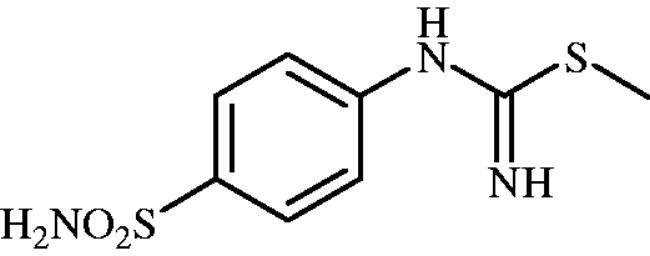


To a solution of 4-thioureidobenzenesulphonamide (**6**) (300 mg, 1.3 mmol) in DMF (4 ml) MeI (0.08 ml, 1.3 mmol) at room temperature was added and the mixture was heated at 40 °C for 2.5 h. After cooling to room temperature water (30 ml) was added and the mixture was extracted with EtOAc (3 × 20 ml). Combined organic layer was washed with aq. sat. NaHCO_3_ (2 × 20 ml) and aq. sat. NH_4_Cl (20 ml), and dried over Na_2_SO_4_. Solvent evaporation under vacuum afforded **8a** (223 mg, 70%) as white powder.

^1^H NMR (300 MHz, DMSO-d_6_) *δ* = 2.37 (s, 3H), 6.63 (s, 2H), 6.94 (s, 2H), 7.22 (s, 2H), 7.71 (d, 2H, *J =* 8.4 Hz) ppm ^13^C NMR (75 MHz, DMSO-d_6_) *δ* = 14.2, 122.8, 127.7, 138.0, 153.9, 157.0 ppm HRMS (ESI) [M + H]^+^: *m*/*z* calcd for (C_8_H_12_N_3_O_2_S_2_) 246.0371. Found 246.0372.

#### Butyl (4-sulphamoylphenyl)carbamimidothioate (8b)


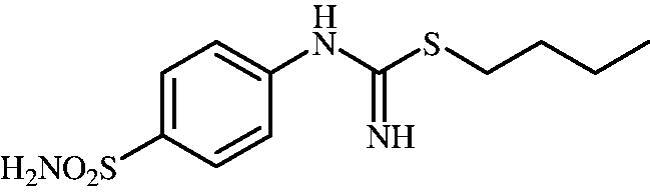


To a solution of 4-thioureidobenzenesulphonamide (**6**) (300 mg, 1.3 mmol) in DMF (4 ml) 1-iodobutane (0.176 ml, 1.6 mmol) at room temperature was added and the mixture was stirred at 30 °C for 24 h. After cooling to room temperature water (30 ml) was added and the mixture was extracted with EtOAc (3 × 20 ml). Combined organic layer was washed with aq. sat. NaHCO_3_ (2 × 20 ml) and aq. sat. NH_4_Cl (20 ml), and dried over Na_2_SO_4_. Solvent evaporation under vacuum afforded **8b** (314 mg, 84%) as a white powder.

^1^H NMR (300 MHz, DMSO-d_6_) *δ* = 0.91 (t, 3H, *J* = 7.2 Hz), 1.33–1.45 (m, 2H), 1.54–1.64 (m, 2H), 2.98 (t, 2H, *J* = 7.2 Hz), 6.59 (s, 2H), 6.92 (d, 2H, *J* = 7.2 Hz), 7.19 (s, 2H), 7.68–7.72 (m, 2H) ppm ^13^C NMR (75 MHz, DMSO-d_6_) *δ* = 13.9, 21.7, 30.0, 31.7, 122.2, 127.2, 137.4, 153.4, 155.5 ppm HRMS (ESI) [M + H]^+^: *m*/*z* calcd for (C_11_H_18_N_3_O_2_S_2_) 288.0840. Found 288.0853.

#### Hexyl (4-sulphamoylphenyl)carbamimidothioate (8c)


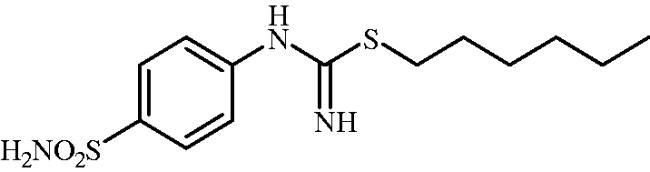


1-Bromohexane (0.181 ml, 1.3 mmol) and potassium iodide (215 mg, 1.3 mmol) were added to DMF (4 ml) and stirred at 100 °C for 1 h to *in situ* generate 1-iodohexane. After cooling to room temperature, 4-thioureidobenzenesulphonamide (**6**) (250 mg, 1.08 mmol) was added to the reaction mixture and the mixture was stirred at 100 °C for 3 h. After cooling to room temperature water (30 ml) was added and the mixture was extracted with EtOAc (3 × 20 ml). Combined organic layer was washed with aq. sat. NaHCO_3_ (2 × 20 ml) and aq. sat. NH_4_Cl (20 ml), and dried over Na_2_SO_4_ and solvent was evaporated under vacuum. The residue was purified by column chromatography on silica gel (DCM:MeOH, 95:5) to afford **8c** (232 mg, 68%) as a white powder.

^1^H NMR (300 MHz, DMSO-d_6_) *δ* = 0.89 (t, 3H, *J* = 6.7 Hz), 1.30–1.42 (m, 6H), 1.56–1.65 (m, 2H), 2.95 (t, 2H, *J* = 7.3 Hz), 6.58 (s, 2H), 6.91 (d, 2H, *J* = 8.3 Hz), 7.19 (s, 2H), 7.71 (d, 2H, *J* = 8.3 Hz) ppm ^13^C NMR (75 MHz, DMSO-d_6_) *δ* = 14.8, 22.9, 28.7, 30.0, 30.8, 31.6, 122.9, 127.6, 137.9, 154.1, 156.0 ppm HRMS (ESI) [M + H]^+^: *m*/*z* calcd for (C_13_H_22_N_3_O_2_S_2_) 316.1153. Found 316.1161.

#### Heptyl (4-sulphamoylphenyl)carbamimidothioate (8d)


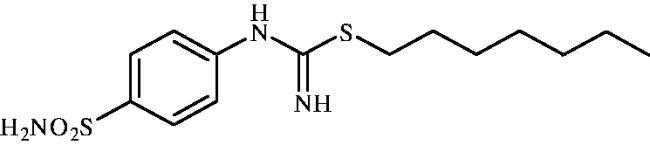


To a solution of 4-thioureidobenzenesulphonamide (**6**) (300 mg, 1.3 mmol) in DMF (4 ml) 1-iodoheptane (0.213 ml, 1.3 mmol) was added at room temperature. The mixture was stirred at 30 °C for 24 h. After cooling to room temperature aq. sat. NaHCO_3_ (20 ml) was added to the reaction mixture. A white precipitate formed was collected by filtration, followed by washing with water and drying on air to afford **8d** (358 mg, 84%) as a white powder.

^1^H NMR (300 MHz, DMSO-d_6_) *δ* = 0.88 (d, 3H, *J* = 6.3 Hz), 1.28 (br. s, 8H), 1.56–1.65 (m, 2H), 2.94 (t, 2H, *J* = 6.9 Hz), 6.59 (s, 2H), 6.92 (d, 2H, *J* = 7.9 Hz), 7.21 (s, 2H), 7.71 (d, 2H, *J* = 7.9 Hz) ppm ^13^C NMR (75 MHz, DMSO-d_6_) *δ* = 14.4, 22.5, 28.5, 28.6, 29.6, 30.3, 31.6, 122.4, 127.2, 137.4, 153.6, 155.5 ppm HRMS (ESI) [M + H]^+^: *m*/*z* calcd for (C_14_H_24_N_3_O_2_S_2_) 330.1310. Found 330.1314.

#### Allyl (4-sulphamoylphenyl)carbamimidothioate (8e)


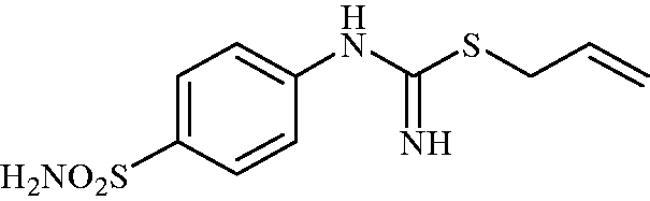


To a solution of 4-thioureidobenzenesulphonamide (**6**) (300 mg, 1.3 mmol) in DMF (4 ml) allyl bromide (0.112 ml, 1.3 mmol) was added and the mixture was stirred at 30 °C for 24 h. After cooling to room temperature water (30 ml) was added and the mixture was extracted with EtOAc (3 × 20 ml). Combined organic layer was washed with aq. sat. NaHCO_3_ (2 × 20 ml) and aq. sat. NH_4_Cl (20 ml), and dried over Na_2_SO_4_. Solvent evaporation under vacuum afforded **8e** (304 mg, 87%) a white powder.

^1^H NMR (300 MHz, DMSO-d_6_) *δ* = 3.66 (d, 2H, *J* = 6.7 Hz), 5.13 (d, 1H, *J* = 9.9 Hz), 5.28 (d, 1H, *J* = 16.9 Hz), 5.85–5.99 (m, 1H), 6.64 (s, 2H), 6.95 (s, 2H), 7.20 (s, 2H), 7.72 (d, 2H, *J* = 8.5 Hz) ppm ^13^C NMR (75 MHz, DMSO-d_6_) *δ* = 33.7, 118.5, 122.8, 127.7, 134.9, 138.0, 153.7, 155.3 ppm HRMS (ESI) [M + H]^+^: *m*/*z* calcd for (C_10_H_14_N_3_O_2_S_2_) 272.0527. Found 272.0538.

#### Prop-2-yn-1-yl (4-sulphamoylphenyl)carbamimidothioate (8f)


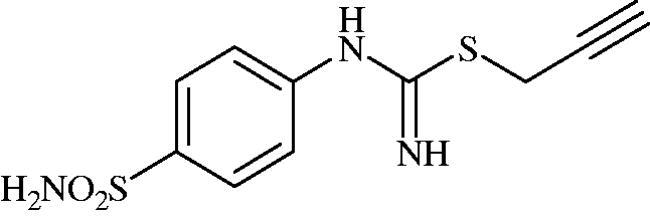


To a solution of 4-thioureidobenzenesulphonamide (**6**) (300 mg, 1.3 mmol) in DMF (4 ml) 3-bromoprop-1-yne (80% in toluene) (0.144 ml, 1.3 mmol) was added at 20 °C and the mixture was stirred at 20 °C for 7 h. After cooling to room temperature water (30 ml) was added and the mixture was extracted with EtOAc (3 × 20 ml). Combined organic layer was washed with aq. sat. NaHCO_3_ (2 × 20 ml) and aq. sat. NH_4_Cl (20 ml), and dried over Na_2_SO_4_. Solvent evaporation under vacuum afforded **8f** (216 mg, 62%) as a white powder.

^1^H NMR (300 MHz, DMSO-d_6_) *δ* = 3.21 (s, 1H), 3.85 (s, 2H), 6.76 (s, 2H), 6.94 (d, 2H, *J* = 6.6 Hz), 7.23 (s, 2H), 7.72 (dd, 2H, *J* = 6.6, 1.5 Hz) ppm ^13^C NMR (75 MHz, DMSO-d_6_) *δ* = 19.2, 74.5, 81.2, 123.0, 127.7, 138.2, 153.5, 154.5 ppm HRMS (ESI) [M + H]^+^: *m*/*z* calcd for (C_10_H_12_N_3_O_2_S_2_) 270.0371. Found 270.0374.

#### Benzyl (4-sulphamoylphenyl)carbamimidothioate (8g)


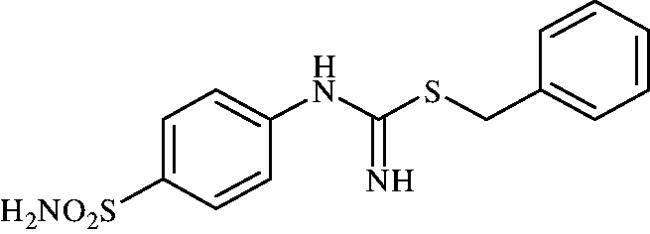


To a solution of 4-thioureidobenzenesulphonamide (**6**) (300 mg, 1.3 mmol) in DMF (4 ml) benzyl bromide (0.153 ml, 1.3 mmol) was added at room temperature and the mixture was stirred at 40 °C for 3 h. After cooling to room temperature water (30 ml) was added and the mixture was extracted with EtOAc (3 × 20 ml). Combined organic layer was washed with aq. sat. NaHCO_3_ (2 × 20 ml) and aq. sat. NH_4_Cl (20 ml), and dried over Na_2_SO_4_. Solvent evaporation under vacuum afforded **8g** (370 mg, 89%) as a white powder.

^1^H NMR (300 MHz, DMSO-d_6_) *δ* = 4.25 (s, 2H), 6.71 (s, 2H), 6.91 (d, 2H, *J* = 8.3 Hz), 7.21 (s, 2H), 7.28–7.41 (m, 5H), 7.71 (d, 2H, *J* = 8.3 Hz) ppm ^13^C NMR (75 MHz, DMSO-d_6_) *δ* = 35.0, 122.8, 127.7, 127.9, 129.3, 129.8, 138.1, 138.9, 153.2, 155.8 ppm HRMS (ESI) [M + H]^+^: *m*/*z* calcd for (C_14_H_16_N_3_O_2_S_2_) 322.0684. Found 322.0693.

#### 4-Chlorobenzyl (4-sulphamoylphenyl)carbamimidothioate(8h)


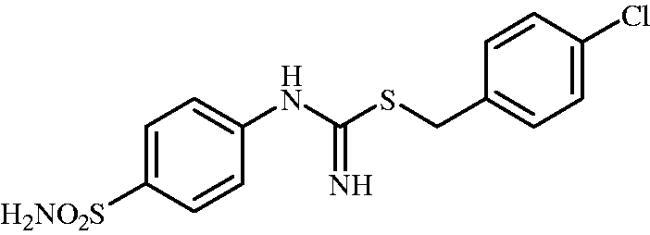


To a solution of 4-thioureidobenzenesulphonamide (**6**) (300 mg, 1.3 mmol) in DMF (4 ml) 4-chlorobenzyl bromide (266 mg, 1.3 mmol) was added at room temperature and the mixture was stirred at 30 °C for 6 h. After cooling to room temperature water (30 ml) was added and the mixture was extracted with EtOAc (3 × 20 ml). Combined organic layer was washed with aq. sat. NaHCO_3_ (2 × 20 ml) and aq. sat. NH_4_Cl (20 ml), and dried over Na_2_SO_4_. Solvent evaporation under vacuum afforded **8h** (271 mg, 80%) as a white powder.

^1^H NMR (300 MHz, DMSO-d_6_) *δ* = 4.25 (s, 2H), 6.80 (s, 2H), 6.96–6.98 (m, 2H), 7.23 (s, 2H), 7.39–7.45 (m, 4H), 7.72 (d, 2H, *J* = 8.5 Hz) ppm ^13^C NMR (75 MHz, DMSO-d_6_) *δ* = 34.0, 122.8, 127.7, 129.2, 131.6, 132.5, 138.1, 138.4, 153.5, 155.5 ppm HRMS (ESI) [M + H]^+^: *m*/*z* calcd for (C_14_H_15_N_3_O_2_S_2_Cl) 356.0294. Found 356.0301.

#### 4-Bromobenzyl (4-sulphamoylphenyl)carbamimidothioate (8i)


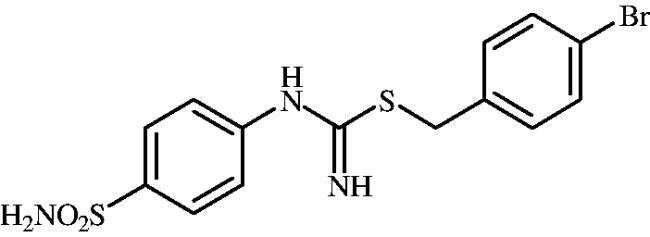


To a solution of 4-thioureidobenzenesulphonamide (**6**) (300 mg, 1.3 mmol) in DMF (4 ml) 4-bromobenzyl bromide (324 mg, 1.3 mmol) was added at room temperature and the mixture was stirred at 30 °C for 6 h. After cooling to room temperature water (30 ml) was added and the mixture was extracted with EtOAc (3 × 20 ml). Combined organic layer was washed with aq. sat. NaHCO_3_ (2 × 20 ml) and aq. sat. NH_4_Cl (20 ml), and dried over Na_2_SO_4_. Solvent evaporation under vacuum afforded **8i** (430 mg, 83%) as a white powder.

^1^H NMR (300 MHz, DMSO-d_6_) *δ* = 4.24 (s, 2H), 6.81 (s, 2H), 6.95–6.98 (m, 2H), 7.23 (s, 2H), 7.37 (d, 2H, *J* = 8.3 Hz), 7.55 (d, 2H, *J* = 8.3 Hz), 7.71–7.74 (m, 2H, m) ppm ^13^C NMR (75 MHz, DMSO-d_6_) *δ* = 34.0, 121.0, 122.8, 127.7, 132.0, 132.1, 138.1, 138.9, 153.4, 155.5 ppm HRMS (ESI) [M + H]^+^: *m*/*z* calcd for (C_14_H_15_N_3_O_2_S_2_Br) 399.9789. Found 399.9798.

#### 4-Cyanobenzyl (4-sulphamoylphenyl)carbamimidothioate(8j)


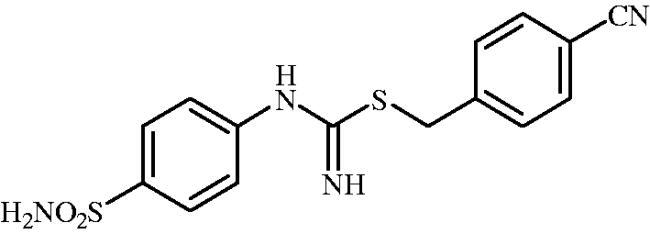


To a solution of 4-thioureidobenzenesulphonamide (**6**) (300 mg, 1.3 mmol) in DMF (4 ml) 4-cyanobenzyl bromide (255 mg, 1.3 mmol) was added at room temperature and the mixture was stirred at 30 °C for 6 h. After cooling to room temperature water (30 ml) was added and the mixture was extracted with EtOAc (3 × 20 ml). Combined organic layer was washed with aq. sat. NaHCO_3_ (2 × 20 ml) and aq. sat. NH_4_Cl (20 ml), and dried over Na_2_SO_4_ and solvent was evaporated under vacuum. The residue was purified by column chromatography on silica gel (DCM:MeOH, 95:5) to afford **8j** (401 mg, 89%) as a white powder.

^1^H NMR (300 MHz, DMSO-d_6_) *δ* = 4.33 (s, 2H), 6.75 (s, 2H), 6.91 (d, 2H, *J* = 8.2 Hz), 7.24 (s, 2H), 7.61 (d, 2H, *J* = 8.2 Hz), 7.72 (d, 2H, *J* = 8.1 Hz), 7.82 (d, 2H, *J* = 8.2 Hz) ppm ^13^C NMR (75 MHz, DMSO-d_6_) *δ* = 34.3, 110.5, 119.8, 122.9, 127.8, 130.8, 133.2, 138.2, 145.7, 153.7, 155.1 ppm HRMS (ESI) [M + H]^+^: *m*/*z* calcd for (C_15_H_15_N_4_O_2_S_2_) 347.0636. Found 347.0645.

#### 4-Nitrobenzyl (4-sulphamoylphenyl)carbamimidothioate(8k)


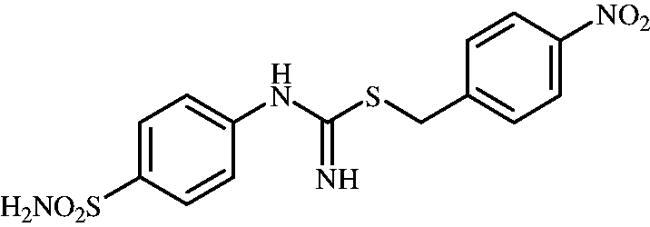


To a solution of 4-thioureidobenzenesulphonamide (**6**) (300 mg, 1.3 mmol) in DMF (4 ml) 4-nitrobenzyl bromide (280 mg, 1.3 mmol) was added at room temperature and the mixture was stirred at 30 °C for 6 h. After cooling to room temperature water (30 ml) was added and the mixture was extracted with EtOAc (3 × 20 ml). Combined organic layer was washed with aq. sat. NaHCO_3_ (2 × 20 ml) and aq. sat. NH_4_Cl (20 ml), and dried over Na_2_SO_4_. Solvent evaporation under vacuum afforded **8k** (390 mg, 82%) as a white powder.

^1^H NMR (300 MHz, DMSO-d_6_) *δ* = 4.39 (s, 2H), 6.79 (s, 2H), 6.95 (s, 2H), 7.24 (s, 2H), 7.67–7.74 (m, 4H), 8.23 (d, 2H, *J* = 8.7 Hz) ppm ^13^C NMR (75 MHz, DMSO-d_6_) *δ* = 34.0, 122.8, 124.4, 127.8, 131.0, 138.2, 147.3, 148.0, 153.4, 155.0 ppm HRMS (ESI) [M + H]^+^: *m*/*z* calcd for (C_19_H_15_N_2_O_2_S_2_) 367.0575. Found 367.0558.

#### 4-(Trifluoromethyl)benzyl (4-sulphamoylphenyl)carbamimidothioate(8l)


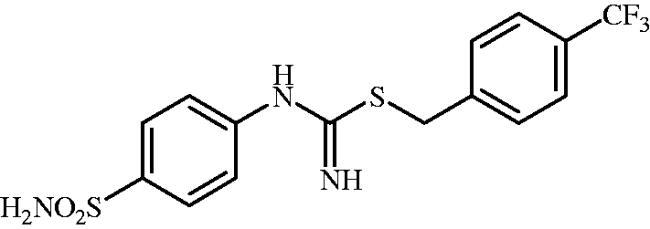


To a solution of 4-thioureidobenzenesulphonamide (**6**) (300 mg, 1.3 mmol) in DMF (4 ml) 1-(bromomethyl)-4-(trifluoromethyl)benzene (310 mg, 1.3 mmol) was added at room temperature and the mixture was stirred at 30 °C for 7 h. After cooling to room temperature water (30 ml) was added and the mixture was extracted with EtOAc (3 × 20 ml). Combined organic layer was washed with aq. sat. NaHCO_3_ (2 × 20 ml) and aq. sat. NH_4_Cl (20 ml), and dried over Na_2_SO and solvent was evaporated under vacuum. The residue was purified by column chromatography on silica gel (DCM:MeOH, 95:5) to afford **8l** (317 mg, 63%) as a white powder.

^1^H NMR (300 MHz, DMSO-d_6_) *δ* = 4.35 (s, 2H), 6.75 (s, 2H), 6.93 (d, 2H, *J* = 7.9 Hz), 7.27 (s, 2H), 7.64 (d, 2H, *J* = 7.9 Hz), 7.71–7.74 (m, 4H) ppm ^13^C NMR (75 MHz, DMSO-d_6_) *δ* = 34.1, 123.0, 126.1 (d, *J* = 3.8 Hz), 127.8, 128.5 (q, *J* = 31.1 Hz), 130.6, 138.2, 144.6, 153.8, 155.2 ppm ^19^F NMR (470 MHz, DMSO-d_6_) *δ* = −60.86 ppm HRMS (ESI) [M + H]^+^: *m*/*z* calcd for (C_15_H_15_N_3_O_2_S_2_F_3_) 390.0558. Found 390.0564.

#### 3,5-Difluorobenzyl (4-sulphamoylphenyl)carbamimidothioate(8m)


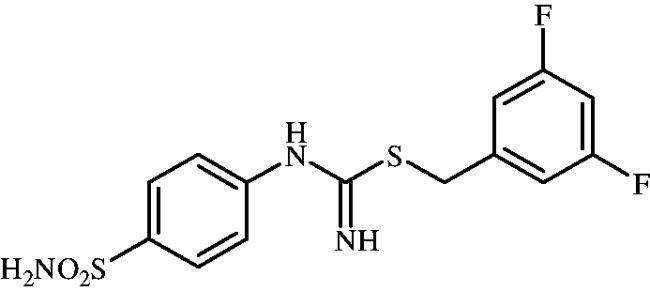


To a solution of 4-thioureidobenzenesulphonamide (**6**) (300 mg, 1.3 mmol) in DMF (4 ml) 1-(bromomethyl)-3,5-difluorobenzene (168 ml, 1.3 mmol) was added at room temperature and the mixture was stirred at 30 °C for 8 h. After cooling to room temperature water (30 ml) was added and the mixture was extracted with EtOAc (3 × 20 ml). Combined organic layer was washed with aq. sat. NaHCO_3_ (2 × 20 ml) and aq. sat. NH_4_Cl (20 ml), and dried over Na_2_SO_4_. Solvent evaporation under vacuum afforded **8m** (395 mg,85%) as a white powder.

^1^H NMR (300 MHz, DMSO-d_6_) *δ* = 4.28 (s, 2H), 6.79 (s, 2H), 6.95 (d, 2H, *J* = 8.5 Hz), 7.13–7.17 (m, 3H), 7.24 (s, 2H), 7.76 (d, 2H, *J* = 8.5 Hz) ppm ^13^C NMR (75 MHz, DMSO-d_6_) *δ* = 33.9, 103.4 (app t, *J* = 26.8 Hz), 112.9 (dd, *J* = 9.5 Hz, 7.7 Hz), 122.8, 127.8, 138.2, 144.4, 153.4, 155.2, 161.1 (dd, *J* = 232.6 Hz, 13.2 Hz) ppm ^19^F NMR(470 MHz, DMSO-d_6_) *δ* = −110.06 ppm HRMS (ESI) [M + H]^+^: *m*/*z* calcd for (C_14_H_14_N_3_O_2_S_2_F_2_) 358.0495. Found 358.0497.

#### 3,5-Dimethylbenzyl (4-sulphamoylphenyl)carbamimidothioate (8n)


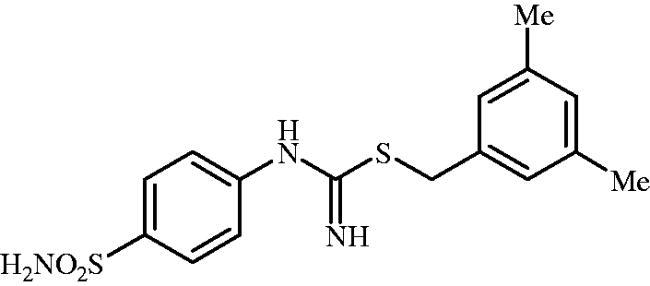


To a solution of 4-thioureidobenzenesulphonamide (**6**) (300 mg, 1.3 mmol) in DMF (4 ml) 1-(bromomethyl)-3,5-dimethylbenzene (261 mg, 1.3 mmol) was added at room temperature and the mixture was stirred at 30 °C for 8 h. After cooling to room temperature water (30 ml) was added and the mixture was extracted with EtOAc (3 × 20 ml). Combined organic layer was washed with aq. sat. NaHCO_3_ (2 × 20 ml) and aq. sat. NH_4_Cl (20 ml), and dried over Na_2_SO_4_ and solvent was evaporated under vacuum. The residue was purified by column chromatography on silica gel (DCM:MeOH, 95:5) to afford **8n** (294 mg, 65%) as a white powder.

^1^H NMR (300 MHz, DMSO-d_6_) *δ* = 2.27 (s, 6H), 4.17 (s, 2H), 6.69 (s, 2H), 6.90–6.93 (m, 3H), 6.98 (s, 2H), 7.21 (s, 2H), 7.70–7.73 (m, 2H) ppm ^13^C NMR (75 MHz, DMSO-d_6_) *δ* = 21.3, 34.4, 122.4, 127.1, 127.2, 128.9, 137.5, 137.8, 138.0, 153.5, 155.2 ppm HRMS (ESI) [M + H]^+^: *m*/*z* calcd for (C_16_H_20_N_3_O_2_S_2_) 350.0997. Found 350.1007.

#### 3,4-Dichlorobenzyl (4-sulphamoylphenyl)carbamimidothioate (8o)


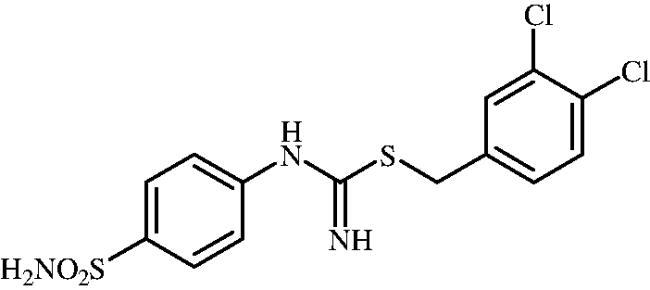


To a solution of 4-thioureidobenzenesulphonamide (**6**) (300 mg, 1.3 mmol) in DMF (4 ml) 4-(bromomethyl)-1,2-dichlorobenzene (189 ml, 1.3 mmol) was added at room temperature and the mixture was stirred at 30 °C for 6 h. After cooling to room temperature water (30 ml) was added and the mixture was extracted with EtOAc (3 × 20 ml). Combined organic layer was washed with aq. sat. NaHCO_3_ (2 × 20 ml) and aq. sat. NH_4_Cl (20 ml), and dried over Na_2_SO_4_ and solvent was evaporated under vacuum. The residue was purified by column chromatography on silica gel (DCM:MeOH, 95:5) to afford **8o** (391 mg, 77%) as a white powder.

^1^H NMR (300 MHz, DMSO-d_6_) *δ* = 4.25 (s, 2H), 6.75 (s, 2H), 6.92 (d, 2H, *J* = 8.0 Hz), 7.24 (s, 2H), 7.41 (d, 1H, *J* = 8.2 Hz), 7.62 (d, 1H,*J* = 8.2 Hz), 7.69 (s, 1H), 7.73 (d, 2H, *J* = 8.0 Hz) ppm ^13^C NMR (75 MHz, DMSO-d_6_) *δ* = 33.4, 122.9, 127.8, 130.2, 130.4, 131.4, 131.6, 131.7, 138.2, 141.1, 153.7, 155.1 ppm HRMS (ESI) [M + H]^+^: *m*/*z* calcd for (C_14_H_14_N_3_O_2_S_2_Cl_2_) 389.9904. Found 389.9911.

### Ca inhibitory assay

An applied photophysics stopped-flow instrument has been used for assaying the CA-catalysed CO_2_ hydration activity^19^. Phenol red (at a concentration of 0.2 mM) was used as indicator, working at the absorbance maximum of 557 nm, with 20 mM Hepes (pH 7.5) as buffer for α-CAs or 20 mM TRIS (pH 8.4) as buffer for β-CAs, and 20 mM Na_2_SO_4_ (for maintaining constant the ionic strength), following the initial rates of the CA-catalysed CO_2_ hydration reaction for a period of 10 – 100 s. The CO_2_ concentrations ranged from 1.7 to 17 mM for the determination of the kinetic parameters and inhibition constants. For each inhibitor, at least six traces of the initial 5 – 10% of the reaction have been used for determining the initial velocity. The uncatalysed rates were determined in the same manner and subtracted from the total observed rates. Stock solutions of inhibitor (0.1 mM) were prepared in distilled – deionised water, and dilutions up to 0.01 nM were done thereafter with the assay buffer. Inhibitor and enzyme solutions were preincubated together for 6 h at room temperature prior to assay in order to allow for the formation of the E – I complex. The inhibition constants were obtained by nonlinear least-squares methods using PRISM 3 and the Cheng–Prusoff equation, as reported earlier^12^^,^^13^^,^^20^^,^^21^, and represent the mean from at least three different determinations. All CA isoforms were recombinant ones obtained in-house as reported earlier^22^^,^^23^ and their concentrations in the assay system ranged between 7.6–12.5 nM.

## Conclusions

A new series of novel alkyl/benzyl (4-sulphamoylphenyl)carbamimidothioates (**8a–o**) was designed, synthesised and evaluated for their ability to inhibit four pharmacologically significant cytosolic hCA isozymes, hCA I, hCA II, hCA VII and hCA XIII as well as three β-CAs from the bacterial pathogens *Mammaliicoccus* (*Staphylococcus*) *sciuri*, MscCA, and *Salmonella enterica* (*serovar Typhimurium*), StCA1 and StCA2. Listed below are some important information of the bioassays: (i) all investigated compounds **8a–o** showed strong inhibitory activities against hCA I (K_Is_, 38.5–92.5 nM) compared to that of AAZ (K_I_, 250.0 nM); (ii) up to half of the compounds investigated here (**8c–f**, **8k–n**) showed better inhibitory activities against hCA II (K_Is_, 1.7–9.4 nM) in comparison with AAZ (K_I_, 12.5 nM); (iii) Among the 15 compounds examined, 8 of them (**8c**, **8e**, **8g–8j**, **8l**, and **8m**) showed better inhibitory activities against hCA VII (K_Is_, 1.2–2.4 nM) in comparison with AAZ (K_I_, 2.5 nM). Importantly, among these 8 compounds, half (**8g–8j**) showed more than 10 times much better selectivity against hCA VII vs hCA II; (iv) All investigated compounds (**8a-o**) exhibited weaker inhibitory activity (K_Is_, 69.3–925.9 nM) than AAZ (K_I_, 16.0 nM); (v) Against the bacterial β-CA isoform MscCA, except compounds **8g** and **8h** with K_I_ values of 360.8 and 618.9 nM, respectively, other compounds acted as weak inhibitors (K_Is_, 658.8–8761 nM) compared to AAZ (K_I_, 625 nM); (vi) Against the bacterial β-CA isoform StCA1, only benzyl- and 4-cyanobenzyl-substituted carbamimidothioates **8g** and **8j** with K_I_ values of 52.2 and 55.7 nM, respectively, showed better inhibitory activities compared to AAZ (K_I_, 59 nM); and (vii) These compounds were generally poor inhibitors of StCA2 with K_I_ values ranging between 270.2 and 9697 nM.
